# Targeted A-to-G base editing of chloroplast DNA in plants

**DOI:** 10.1038/s41477-022-01279-8

**Published:** 2022-12-01

**Authors:** Young Geun Mok, Sunghyun Hong, Su-Ji Bae, Sung-Ik Cho, Jin-Soo Kim

**Affiliations:** 1 Center for Genome Engineering, Institute for Basic Science, Daejeon, Republic of Korea; 2 Department of Chemistry, Seoul National University, Seoul, Republic of Korea; 3 Present Address: GreenGene Inc., Seoul, Republic of Korea

**Keywords:** Genetic engineering, Molecular engineering in plants

## Abstract

Chloroplast DNA (cpDNA) encodes up to 315 (typically, 120–130) genes^1^, including those for essential components in photosystems I and II and the large subunit of RuBisCo, which catalyses CO_2_ fixation in plants. Targeted mutagenesis in cpDNA will be broadly useful for studying the functions of these genes in molecular detail and for developing crops and other plants with desired traits. Unfortunately, CRISPR–Cas9 and CRISPR-derived base editors, which enable targeted genetic modifications in nuclear DNA, are not suitable for organellar DNA editing^2^, owing to the difficulty of delivering guide RNA into organelles. CRISPR-free, protein-only base editors (including DddA-derived cytosine base editors^3–8^ and zinc finger deaminases^9^), originally developed for mitochondrial DNA editing in mammalian cells, can be used for C-to-T, rather than A-to-G, editing in cpDNA^10–12^. Here we show that heritable homoplasmic A-to-G edits can be induced in cpDNA, leading to phenotypic changes, using transcription activator-like effector-linked deaminases^13^.

## Abstract (web summary)

Plant-optimized transcription activator-like effector-linked deaminases enable site-specific A-to-G base editing in the chloroplast genome, leading to heritable homoplasmic base conversions and phenotypic changes.

## Main

**Fig. 1:**
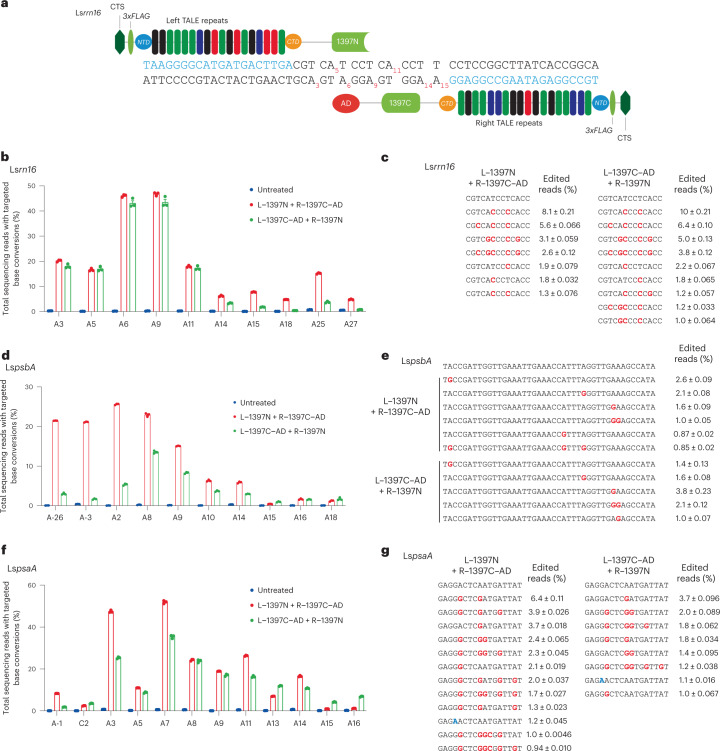
TALED-mediated cpDNA editing in lettuce protoplasts. **a**, TALED pairs designed to target the *rrn16* chloroplast gene in lettuce (*Ls*). The TALE-binding sites are shown in blue. CTS, chloroplasts target signal; NTD, N-terminal domain; CTD, C-terminal domain. **b**–**g**, Frequencies of base conversions and edited alleles induced by TALEDs in *rrn16* (**b**,**c**), *psbA* (**d**,**e**) and *psaA* (**f**,**g**). A-to-G and C-to-T edited bases are shown in red and blue, respectively (**c**,**e**,**g**). The error bars show the standard error of the mean (±s.e.m.) for three biologically independent experiments. Source data

**Extended Data Fig. 1:**
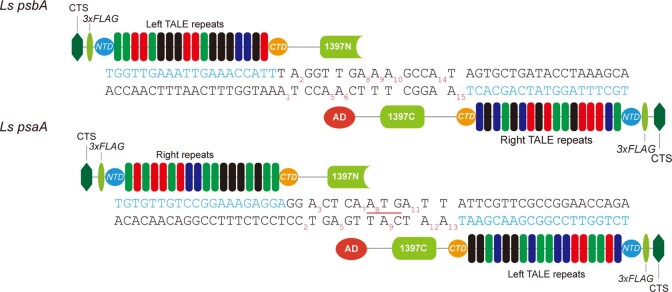
TALED pairs designed to target *psbA* and *psaA* chloroplast genes in lettuce. The TALE-binding sites are shown in blue. CTS, Chloroplasts target signal; NTD, N-terminal domain; CTD, C-terminal domain, 1397 N and 1397 C, split DddA_tox_ half; AD, adenine deaminase (ABE8e).

To demonstrate targeted A-to-G editing in plant organelles using transcription activator-like effector (TALE)-linked deaminases (TALEDs), which are composed of custom-designed TALE DNA-binding arrays, split DddA_tox_ originating from *Burkholderia cenocepacia* and an engineered deoxyadenosine deaminase (TadA-8e) derived from the *Escherichia coli* TadA protein, we first chose three chloroplast genes (*rrn16*, *psbA* and *psaA*) in lettuce (*Lactuca sativa*) (Fig. 1a and Extended Data Fig. 1). Mutations in these genes give rise to resistance to antibiotics (*rrn16* encoding 16S ribosomal RNA)^14^ or herbicide (*psbA*)^15^ and to an albino phenotype (*psaA*)^12^. We co-transfected in vitro transcripts (mRNA) encoding TALEDs with a plastid transit peptide (PTP) of the *Arabidopsis* RecA1 protein into lettuce protoplasts and measured base editing frequencies using targeted deep sequencing at day 7 post-transfection (Fig. 1b–g). As expected, two TALED pairs (L–1397N (left-side TALE fused to the amino-terminal half of DddA_tox_ split at Gly1397) + R–1397C–AD (right-side TALE fused to the carboxy-terminal half of DddA_tox_ split at Gly1397 and the TadA-8e adenine deaminase) and L–1397C–AD + R–1397N) targeted to the *rrn16* site induced A-to-G conversions with editing frequencies of up to 46% without causing C-to-T conversions (Fig. 1b,c). Adenines in the spacer region between the two TALE-binding sites were edited more efficiently than those positioned outside the region. Two TALED pairs (L–1397N + R–1397C–AD and L–1397C–AD + R–1397N) targeted to the *psbA* site also induced A-to-G conversions with editing frequencies of up to 25%. Bystander A-to-G edits (A-26) were also induced outside of the spacer region with a frequency of 21% by the L–1397N + R–1397C–AD pair (Fig. 1d), which could have been caused by relatively poor affinity of the left-side TALE array (L) for the target DNA. At the *psaA* target site, two TALED pairs (L–1397N + R–1397C–AD and L–1397C–AD + R–1397N) induced A-to-G conversions in lettuce protoplasts with editing frequencies of up to 51% (Fig. 1f). Unexpectedly, C-to-T conversions (C2) were also induced with up to 3.5% efficiencies (Fig. 1f), suggesting that uracil-glycosylase-inhibitor-free split TALEDs could induce C-to-T edits in addition to A-to-G edits (albeit less efficiently) in chloroplasts, unlike in mammalian mitochondria.

**Fig. 2:**
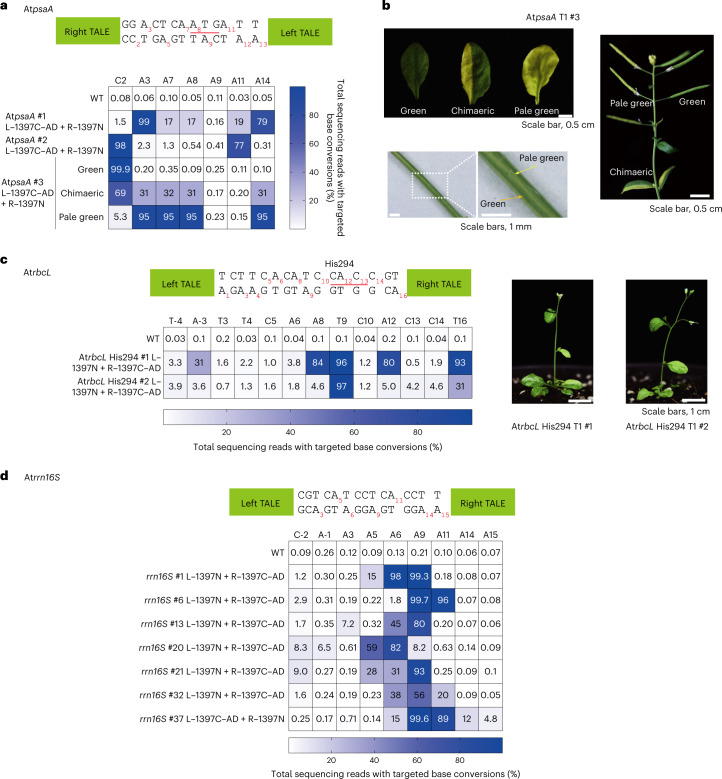
TALED-mediated cpDNA editing in *Arabidopsis*. **a**–**d**, Heat maps showing editing frequencies and phenotypes of *Arabidopsis* T1 plants expressing TALEDs targeted to *psaA* (**a**,**b**), *rbcL* (**c**) and *rrn16S* (**d**). The TALE-binding regions are shown in green. The start codon in *psaA* (**a**) and the codon for His294 in *rbcL* (**c**) are underlined. WT, wild type. Source data

**Extended Data Fig. 2:**
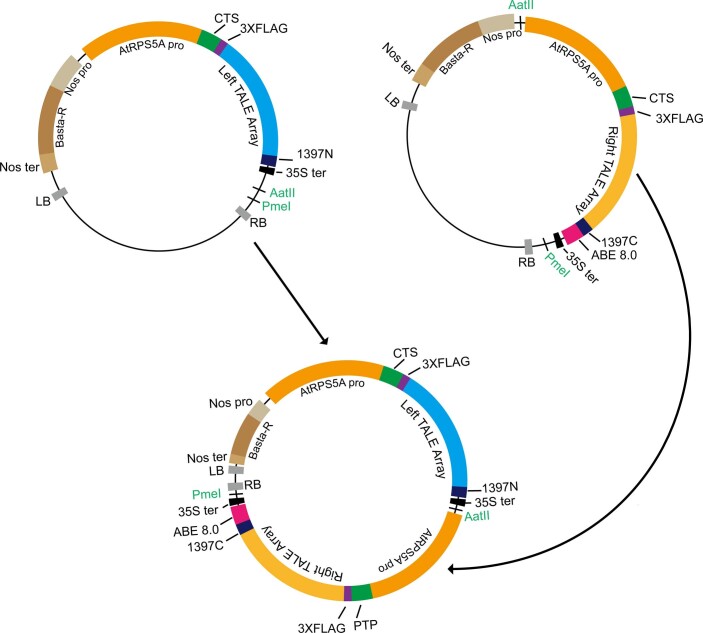
TALED of chloroplast target constructs assembly schematic. Each TALED pairs were cloned into a different T-vector: Left TALE(L-1397N) cloned into RPS5A_pro_-CTS-35S_ter_-Aat II-Pme I T-vector and Right TALE (R-1397C-AD) cloned into Aat II-RPS5A_pro_- CTS-35S_ter_-PmeI. After digesting each vector with Aat II and Pme I, Aat II-RPS5A_pro_-CTS-Right TALE-1397C-AD-35S_ter_-PmeI was ligated to a vector with pRPS5A_pro_-CTS-Left TALE-1397N-35S_ter_-Aat II-Pme I, and the TALED pair was cloned into one vector. 1397 N and 1397C-ABE8.0 (ABE8e), which intersected in Left TALE and Right TALE, were also constructed.

**Extended Data Fig. 3:**
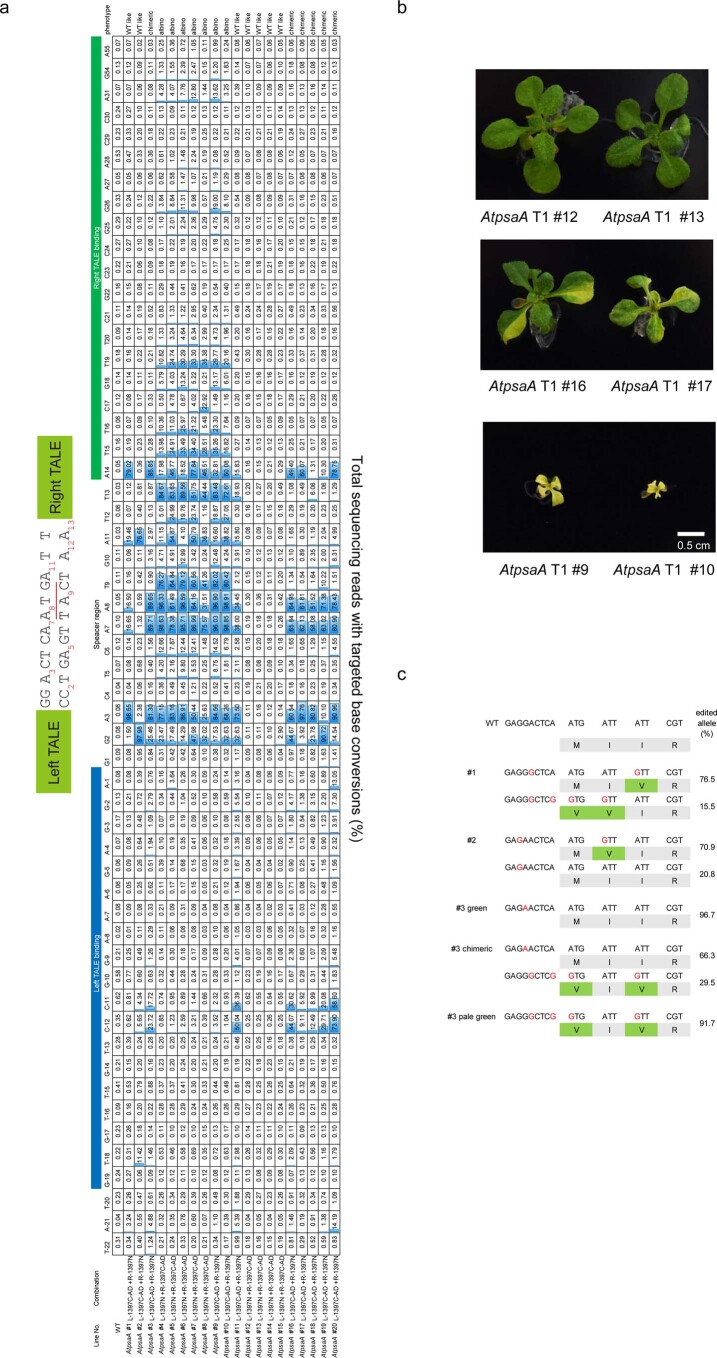
Analysis of *AtpsaA* T1 plants. Editing frequencies (a) and phenotypes (b) and allele frequencies (c) of *psaA* targeted T1 plants. The TALE binding regions are shown in green, and analyzed nucleotides are shown in numbers. The adenine of the desired target for base editing is indicated in red underline. Edited bases were represented by red (c). The base conversion rates were measured by targeted deep sequencing. Source data

We next chose three *Arabidopsis* chloroplast genes (*psaA*, *rbcL* and *rrn16S*) to investigate whether TALED-mediated edits in chloroplast DNA (cpDNA) could be stably maintained in whole plants. We obtained T1 transformants using a transfer DNA binary vector encoding a TALED pair specific to each gene under the control of the *RPS5A* promoter and the 35S terminator^11^ (Extended Data Fig. 2). Targeted deep sequencing showed that A-to-G base editing was induced at multiple positions with frequencies of up to 99% in 16 of 20 T1 plants transformed with the *psaA*-targeted TALED pair (Extended Data Fig. 3a). Among the 16 edited transformants, 15 plants had mutations that disrupted the start codon (A8 and A9, underlined in Fig. 2a). As a result, seven transformants showed an albino phenotype to varying degrees. Two plantlets with almost complete albino phenotypes failed to grow (Extended Data Fig. 3b). As shown with lettuce protoplasts (Fig. 1f,g), C-to-T conversions were also observed, with editing frequencies of up to 98% (#2, C2) in these T1 plants (Extended Data Fig. 3c). Interestingly, unlike *psaA* T1 #1 and #2 plants showing a wild-type morphology without an albino phenotype, the *psaA* T1 #3 plant was mosaic, with green, chimaeric and pale-green leaves. Pale-green traits were also observed in stems and siliques (Fig. 2b). In particular, the editing frequencies of an adenine (A8) in the start codon were 0.1%, 31% and 95% in green, chimaeric and pale-green leaves, suggesting that albinism was caused by the disruption of the *psaA* gene via adenine base editing (Fig. 2a,b). The other five chimaeric T1 plants (#16–20) also showed A-to-G conversions at this position (A8), with frequencies that ranged from 52% to 78% (Extended Data Fig. 3a). Two T1 (#9 and #10) plantlets with the highest editing frequencies (97% and 99%) at this position died prematurely. These results demonstrate that nearly homoplasmic (~99%) adenine editing can be obtained in *Arabidopsis* T1 transformants using TALEDs expressed under the control of the *RPS5A* promoter, which is active at an early embryonic stage in meristem regions^16^.

**Extended Data Fig. 4:**
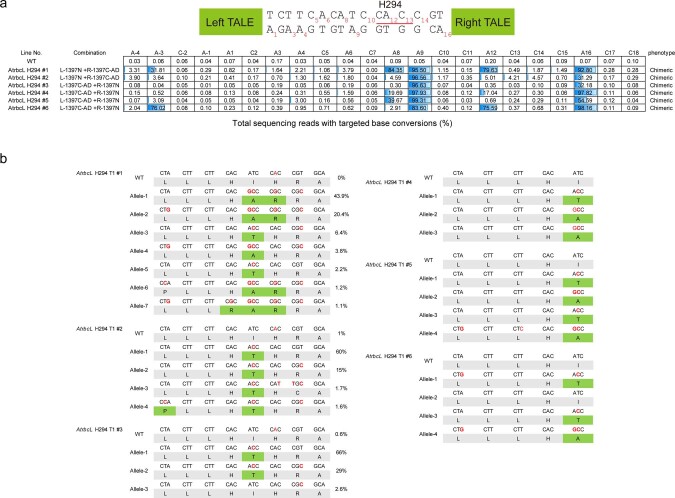
Analysis of *AtrbcL* T1 plants. Editing frequencies (a) and allele frequencies (b) of *rbcL* targeted T1 plants. The TALE binding regions are shown in green, and analyzed nucleotides are shown in numbers. The adenine of the desired target for base editing is indicated in red underline. Edited bases were represented by red (b). The base conversion rates were measured by targeted deep sequencing. Source data

**Extended Data Fig. 5:**
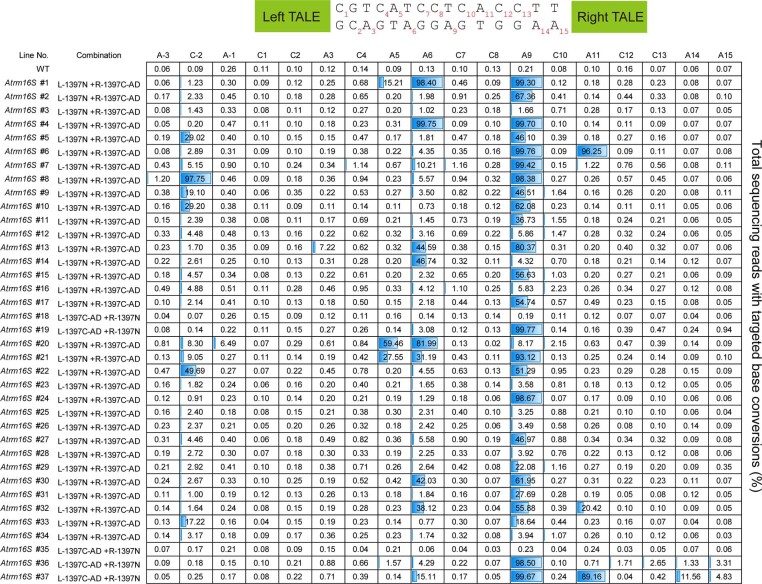
Analysis of *Atrrn16S* T1 plants. Editing frequencies of *rrn16S* targeted T1 plants. The TALE binding regions are shown in green, and analyzed nucleotides are shown in numbers. The base conversion rates were measured by targeted deep sequencing. Source data

We next analysed *rbcL*-targeted T1 plants. The *rbcL* gene encodes the large, catalytic subunit of RuBisCo, the enzyme catalysing CO_2_ fixation in chloroplasts^1^. A-to-G conversions were induced at several positions in the spacer region with editing frequencies of up to 99.3% in a total of six T1 transformants with chimaeric leaves (Fig. 2c and Extended Data Fig. 4). C-to-T conversions were rarely induced at two positions in a 5′-TC-3′ context (C5 and C10) in these plants. These mutations gave rise to alterations in amino acid sequences around His294 (underlined in Fig. 2c), an amino acid residue absolutely conserved among all members of the RuBisCo superfamily. Apparently, the resulting RuBisCo proteins were poorly active, resulting in partial albinism. In addition, we obtained a total of 37 T1 plants with the *rrn16S*-targeted TALED. Among these, 35 plants were base edited with frequencies that ranged from 1.3% to 99.8% (Fig. 2d and Extended Data Fig. 5), demonstrating that homoplasmic mutations can be induced in whole plants using TALEDs.

**Fig. 3:**
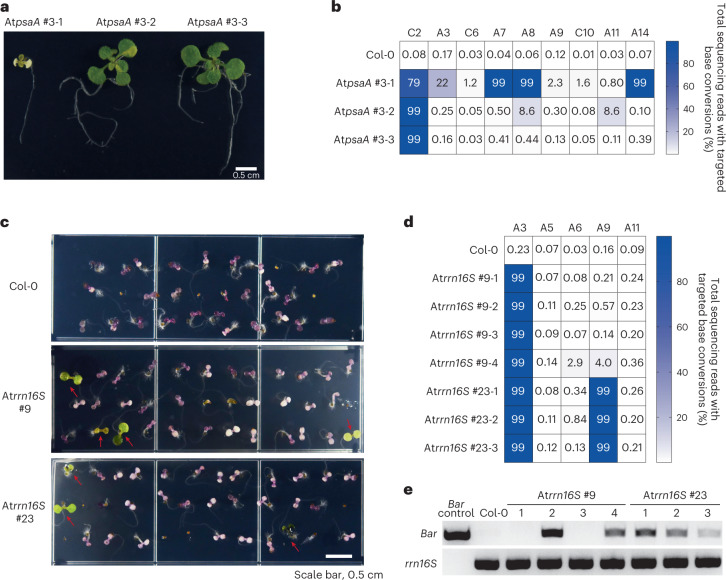
Phenotypes and genotypes of *Arabidopsis* T2 plants. **a**, Two-week-old T2 seedlings from the #3 *psaA*-edited T1 plant grown on half-strength MS medium containing 1% sucrose under long-day conditions. **b**, Base-editing frequencies of T2 progenies. **c**, One-week-old Col-0 and T2 seedlings from At*rrn16S*-edited T1 plants (#9 and #23) grown on half-strength MS medium containing 1% sucrose and spectinomycin (10 mg l^−1^) under long-day conditions. The red arrows indicate spectinomycin-resistant seedlings. **d**, Base-editing frequencies of spectinomycin-resistant At*rrn16S*-edited T2 progenies. **e**, PCR amplification of the Bar transgene (552 base pairs) in genomic DNA isolated from spectinomycin-resistant T2 seedlings. *rrn16S* (235 base pairs) was used as an internal control. Source data

**Extended Data Fig. 6:**
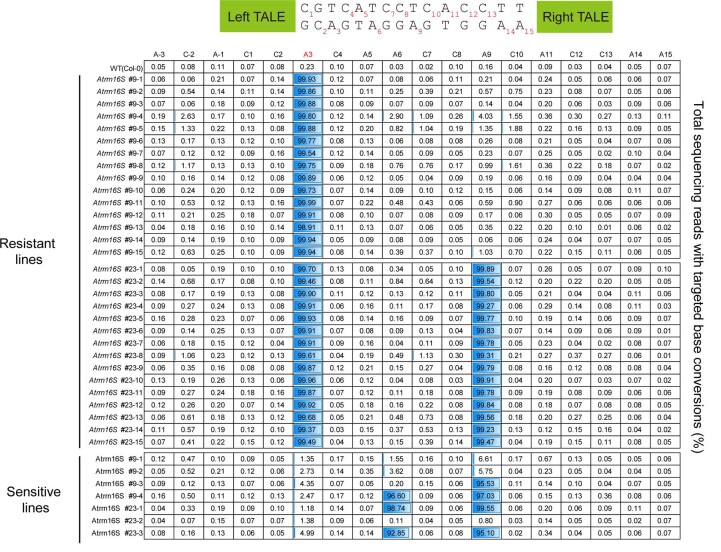
Analysis of *Atrrn16S* T2 plants. Editing frequency of *rrn16S*-targeted T2 plants with spectinomycin (10 mg l^-1^) resistance and sensitivity. The TALE binding regions are shown in green, and analyzed nucleotides are shown in numbers. The base conversion rates were measured by targeted deep sequencing. Source data

Next, we investigated whether TALED-induced edits in cpDNA were inherited in the next generation. We harvested T2 seeds from the #3 *psaA*-edited T1 plant and grew T2 progenies on half-strength Murashige and Skoog (MS) media under long-day (16 h of light and 8 h of dark) conditions for 14 days to confirm phenotypic alterations. Interestingly, T2 plants showed diverse phenotypes and genotypes (Fig. 3a,b). Targeted deep sequencing showed that A8 in the start codon was almost completely (99%), partially (8.6%) and poorly (0.44%) edited in albino (#3-1), chimaeric (#3-2) and wild-type-like (#3-3) plants, respectively (Fig. 3b), suggesting that the disruption of the initiation codon gave rise to albinism. Furthermore, we were able to obtain spectinomycin-resistant plants after T2 seeds of *rrn16S*-edited T1 lines were sown in half-strength MS medium containing spectinomycin, an antibiotic that inhibits protein synthesis (Fig. 3c). Interestingly, A3, which had been minimally edited in T1 plants with an average editing frequency of 0.47 ± 0.19%, was almost completely (99%) converted to guanine in several T2 plants resistant to spectinomycin (Fig. 3d). No other mutations, in addition to A3-to-G conversions, were induced at >1.0% frequencies in a total of 13 spectinomycin-resistant, *rrn16S-*edited #9 T2 lines (Extended Data Fig. 6), suggesting that A3 editing was responsible for the drug resistance. We also found that two spectinomycin-resistant T2 plants (#9-1 and 3) were transgene-free (Fig. 3e), indicating that the A3-to-G edit was induced in T1 plants and that the insertion of multiple copies of transgene is not required to induce homoplasmic editing. Note also that transgene-free, cpDNA-edited plants can be exempt from genetically modified organism regulations^17,18^. Taken together, these results demonstrate that base edits induced by TALEDs in cpDNA are transmitted to the next generation.

**Fig. 4:**
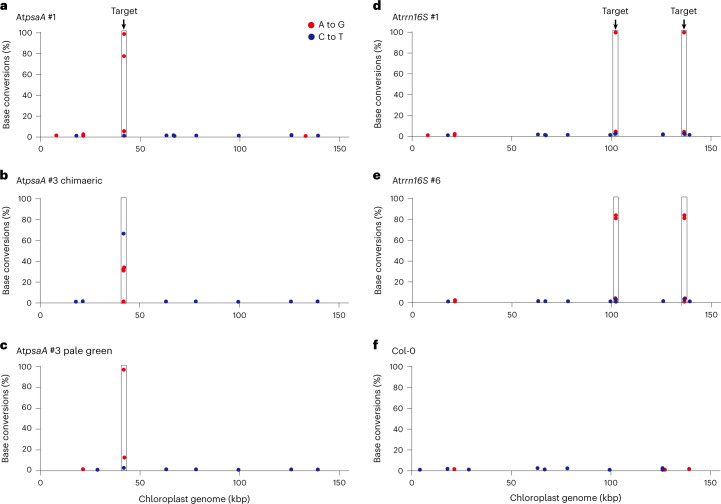
Analysis of TALED-induced off-target effects in the *Arabidopsis* plastid genome. **a**–**f**, Chloroplast-genome-wide plots showing A-to-G or C-to-T base conversions relative to the reference genome (AP000423.1). We analysed *psaA*-edited (**a**–**c**) and *rrn16S*-edited (**d**,**e**) T1 plants in addition to Col-0 (**f**). Note that there are two identical target sites for the *rrn16S*-specific TALED. kbp, kilobases. Source data

Last, we profiled the off-target activity of the TALEDs targeted to the *psaA* and *rrn16S* sites in T1 plants using whole-genome sequencing (Fig. 4). We were able to confirm on-target edits in each sample and to identify several A-to-G or C-to-T mutations with low conversion frequencies that ranged from 1.0% to 4.4% in the chloroplast genome. Most of these single-nucleotide conversions, relative to the reference chloroplast genome, were also observed in the negative control (Col-0), suggesting that they represent naturally occurring heteroplasmy rather than TALED-induced off-target edits. Note also that a few of the single-nucleotide variations with low heteroplasmic fractions found in Col-0 were not detected in T1 plants or vice versa. These results show that TALEDs induce on-target edits specifically without substantial unlinked off-target mutations in cpDNA.

The TALED-mediated organellar base editing described in this study has a number of advantages over plastid transformation via homologous recombination using a targeting vector. First, organellar base editing is more broadly applicable, not limited to certain plant species amenable for plastid transformation. Second, homoplasmic editing can be achieved in a single round of *Agrobacterium*-mediated transformation, as shown in this study. In contrast, multiple rounds of marker selection and regeneration are required to obtain homoplasmy using plastid transformation. Third, no transgenes are integrated in plastid DNA with organellar base editing. In contrast, plastid transformation involves the targeted integration of a selectable marker gene into plastid DNA, which cannot be removed by breeding.

In summary, we developed plant-optimized TALEDs for A-to-G base editing in cpDNA in protoplasts and whole plants. In particular, targeted adenine editing in chloroplast genes using *Agrobacterium*-mediated transformation gave rise to heritable homoplasmic mutations, leading to phenotypic changes in *Arabidopsis*. Unlike in human mitochondrial DNA, uracil-glycosylase-inhibitor-free TALEDs catalysed C-to-T (in addition to A-to-G) conversions at some but not all positions in the context of a 5′-TC-3′ motif in cpDNA. It will be important to investigate whether TALEDs yield different editing outcomes in mammalian mitochondria and in plant chloroplasts, possibly because of the differences in mismatch DNA repair systems, and to develop TALEDs catalysing A-to-G conversions exclusively (without C-to-T conversions) in plants. TALEDs, together with DddA-derived cytosine base editors, can now induce targeted A-to-G and C-to-T base editing in plant organellar DNA, which could pave the way for enhancing the efficiency of photosynthesis and CO_2_ fixation in plants, contributing to agricultural innovations and carbon neutralization.

## Methods

### Plasmid construction

TALE arrays were designed to target *rrn16S*, *psaA*, *psbA* and *rbcL* following the approach used in previous reports^4,5,10,13,19^. PCR amplicons encoding the TALE array, DddA_tox_ split and ABE8e, generated using PrimeSTAR GXL DNA Polymerase (TAKARA), were cloned into pRPS5A–CTS digested with SmaI and KpnI (NEB) using Gibson assembly (NEB). Specifically, the left-side and right-side TALED sequences were cloned into pRPS5A–CTS–35S terminator–AatII–PmeI vector and AatII–pRPS5A–CTS–35S terminator–PmeI, respectively. The left-side and right-side TALED constructs were transcribed in vitro, and the resulting mRNAs were used for lettuce protoplast transfection. For *Arabidopsis* transformation, the left and right TALED vectors were digested with AatII and PmeI (NEB) and ligated using T4 DNA ligase (NEB) (Extended Data Fig. 2). The assembled plasmids were chemically transformed into *E. coli* DH5ɑ, and plasmids from the surviving colonies were analysed by the Sanger sequencing method. The TALE binding sequences are listed in Supplementary Table 1. The primers for PCR amplification are listed in Supplementary Table 2. The plasmids used in this study and their annotated DNA sequences are available from Addgene (ID 189639–189652).

### Lettuce protoplast isolation and transfection

Lettuce (*Lactuca sativa* cv. Cheongchima) seeds were sterilized by immersing them in 70% ethanol for 30 s and then in a 0.4% hypochlorite solution for 15 min prior to washing them three times in distilled water. The sterilized lettuce seeds were germinated on half-strength MS medium supplemented with 2% sucrose at 25 °C under 16 h of light and 8 h of dark. Protoplast isolation and transfection were performed as described in a previous report^10^.

### mRNA in vitro transcription

For use as in vitro transcription templates, DNA templates were amplified using PrimeSTAR GXL DNA Polymerase (TAKARA). The mRNAs were synthesized and purified using an in vitro mRNA synthesis kit (Enzynomics). We used 40 μg of each type of transcript for protoplast transfection. The primers for DNA template PCR amplification are listed in Supplementary Table 2.

### Plant transformation and transformant selection

*A. thaliana* Columbia-0 (Col-0) plants were transformed by floral dipping with *Agrobacterium tumefaciens* strain GV3101 as described in a previous report^20^. After transformation, *Arabidopsis* T1 seeds were plated on half-strength MS medium containing 1% sucrose, 20 mg l^−1^ phosphinothricin and 250 mg l^−1^ cefataxim. All transgenic plants were grown at 23 °C under long-day conditions (16 h of light and 8 h of dark).

### Targeted deep sequencing

Total DNA was isolated from cultured cells at day 7 post-transfection^10^ and true leaves from selected plants using a DNeasy Plant Mini kit (Qiagen). On-target sites were amplified through a primary PCR, a secondary PCR and a third PCR to generate deep sequencing libraries using TruSeq HT Dual index-containing primers and PrimeSTAR GXL DNA Polymerase (TAKARA). Illumina MiniSeq paired-end sequencing systems were used to sequence the libraries. The base editing frequencies are presented as percentages of sequencing reads containing base conversions among total sequencing reads. The program used to analyse the frequency of edits is available at https://github.com/ibs-cge/maund. The PCR primer sequences are shown in Supplementary Tables 3 and 4.

### Genotyping of T2 plants

Total DNAs were isolated from true leaves from T2 plants using a DNeasy Plant Mini kit (Qiagen). The regions of interest were amplified from total DNAs using PrimeSTAR GXL DNA Polymerase (TAKARA), after which the PCR amplicons were analysed on a 1% agarose gel. The PCR primer sequences are shown in Supplementary Tables 2 and 4.

### Next-generation sequencing

For analysis of off-target effects, single nucleotide polymorphisms were called in the plastid genomes using sequencing data from total DNA. First, paired-end libraries were prepared from total DNA using a TruSeq DNA PCR-Free Kit (Illumina) for At*psa* #1 and At*rrn16S* #1 and #6 and a TruSeq Nano DNA Kit (Illumina) for At*psa* #3 (chimaeric and pale-green leaf samples) and Col-0. Sequencing was performed with an Illumina HiSeq X Ten platform. To analyse the next-generation sequencing data from whole-chloroplast-genome sequencing, we followed the methods in a previously published report^11^. Paired-end reads were mapped to reference sequences (AP000423.1) using BWA (v.0.7.17) (ref. ^21^) in single-ended mode. We filtered out mapped reads with mapping identities ≤99%. Single nucleotide polymorphisms were then called using pysam (v.0.18.0) (ref. ^22^). Finally, we listed the positions of variants with A-to-G and C-to-T conversion rates ≥1% with read depths ≥5,000.

### Reporting summary

Further information on research design is available in the Nature Portfilio Reporting Summary linked to this article.

## Supplementary information

Supplementary Information Supplementary Tables 1–4 and Sequences 1 and 2.

Reporting Summary

## Data Availability

The DNA sequencing data have been deposited in the National Center for Biotechnology Information (NCBI) Sequence Read Archive (SRA) database with BioProject accession code PRJNA858174. The protein sequences of the TALE arrays and TadA-8e are provided in the Supplementary Information. Any other additional data are available in the Supplementary Information. Source data are provided with this paper.

## Source data

Source Data Fig. 1 Targeted deep sequencing reads.
Source Data Fig. 2 Targeted deep sequencing reads.
Source Data Fig. 3 Targeted deep sequencing reads and unprocessed gel image.
Source Data Fig. 4 Next-generation sequencing depths.
Source Data Extended Data Fig. 3a Targeted deep sequencing reads.
Source Data Extended Data Fig. 4a Targeted deep sequencing reads data.
Source Data Extended Data Fig. 5 Targeted deep sequencing reads data.
Source Data Extended Data Fig. 6 Targeted deep sequencing reads data.

## Code availability

Source code (https://github.com/ibs-cge/maund, created by BotBot Inc.) was used to calculate base editing frequencies from targeted deep sequencing data.
