# Supplementary material for: Targeted A-to-G base editing of chloroplast DNA in plants
Source: Nat Plants. 2022 Dec 1;8(12):1378–84. doi: 10.1038/s41477-022-01279-8 (PMC9788985; doi:10.1038/s41477-022-01279-8)
Supplement: Supplementary file 1 — Supplementary Tables 1–4 and Sequences 1 and 2. [file 41477_2022_1279_MOESM1_ESM.pdf]

nature plants

Letter

https://doi.org/10.1038/s41477-022-01279-8

Targeted A-to-G base editing of chloroplast DNA in plants

In the format provided by the authors and unedited

Supplementary Information

Targeted A-to-G base editing of chloroplast DNA in plants

Young Geun Mok[1,3,4], Sunghyun Hong[1,3,4], Su-Ji Bae[1], Sung-Ik Cho[1 2], and Jin-Soo Kim[1,3]

[1] Center for Genome Engineering, Institute for Basic Science, Daejeon, Republic of Korea.

[2] Department of Chemistry, Seoul National University, Seoul, Republic of Korea.

[3] Present address: GreenGene Inc. Seoul, Republic of Korea.

[4] These authors contributed equally to this work.

Correspondence should be addressed to J.-S.K. (jskim01@snu.ac.kr).

Supplementary Table 1. TALE binding sequences

Supplementary Table 2. PCR primer sequences for cloning, genotype and in vitro transcription templates.

Supplementary Table 3. 1st PCR primer sequences for targeted deep sequencing.

Supplementary Table 4. Genotyping and 2nd PCR primer sequences for targeted deep sequencing.

Supplementary Sequence 1. Amino acid sequences of TALED components.

Supplementary Sequence 2. DNA sequences of RPS5A promoter and 35S terminator.

Supplementary Table 1. TALE binding sequences.

Species	Gene	position	sequence (5` to 3`)
Arabidopsis & Lettuce Sativa	rrn16S & rrn16	Left	taaggggcatgatgacttga
		Right	tgccggtgataagccggagg
Arabidopsis & Lettuce Sativa	psaA	Left	tctggttccggcgaacgaat
		Right	tgtgttgtccggaaagagga
Arabidopsis	rbcL H294	Left	tgccgagataatggcctact
		Right	tatcaataacagcgtgcatt
Lettuce Sativa	psbA	Left	tggttgaaattgaaaccatt
		Right	tgctttaggtatcagcact

Supplementary Table 2. PCR primer sequences for cloning, genotype and in vitro transcription templates.

Name	Sequence (5` to 3`)
TALE F	caaggatgacgatgacaagcccggggatctacgcacgctcggctacagccagcag
TALE 1397 N R	gtagctacctgatccggatccccccaacccctttttcacagcatcc
TALE 1397 C R	gtatggcactgccggatccccccaacccctttttcacagcatcc
G1397N F	ggggttgggggatccggatcaggtagctacgcacttggtcc
G1397N R	ccgcgggatatcgattatcaggtaccctagccttcaggtgggacgacagtc
1397C F	tgaaaaaggggttgggggatccggcagtgccatacctgtgaag
1397C ABE R	catggccgcgggatatcgattatcaggtaccctagttgatggagctctgggccttc
Basta F	tcagatttcggtgacgggca
Basta R	atgagcccagaacgacgc
RPS5A mRNA F	taatacgactcactatagggagagctctgtttctctcaccacagccatggattcacagctagtcttgtctc
RPS5A mRNA R	cggacgtcgcatgcctgcaggtcactggattttgg

Supplementary Table 3. 1st PCR primer sequences for targeted deep sequencing.

Name	Forward sequence (5` to 3`)	Reverse sequence (5` to 3`)
At & Ls 16S rRNA	aacgaattcaccgccgtatg	gctcgcgtctgattagctag
AtpsaA	ccaagtggtagtatcaggtcccttagc	ccccagatcgacttccagatcataattgc
AtrbcL	gccagagaattgggagttcctatcgtaatgc	ccttcaagtttacctactactgtacccgcgtg
LspsbA	gcataacttccataccaaggttagcacgg	catggttattttggccgattgatcttcc
LpsaA	cccggtctagcccattcctcgaatgaag	ctctagtgaataactaaagaaaatagatagatgggag

Supplementary Table 4. Genotyping and 2nd PCR primer sequences for targeted deep sequencing.

Name	Sequence (5` to 3`)
At & Ls 16S rRNA F	acactctttccctacacgacgctcttccgatctgttgcagcctgcaatccgaactgaggac
At & Ls 16S rRNA R	gtgactggagttcagacgtgtgctcttccgatctgtaaggtgttgggttaagtcccgcaacg
AtpsaA F	acactctttccctacacgacgctcttccgatctccaagtggtagtatcaggtcccttagc
AtpsaA R	gtgactggagttcagacgtgtgctcttccgatctaaaattctaagtatctatcatcggttcac
AtrbcL F	acactctttccctacacgacgctcttccgatctgccagagaattgggagttcctatcgtaatgc
AtrbcL R	gtgactggagttcagacgtgtgctcttccgatctccttcaagtttacctactactgtacccgcgtg
LspsbA F	acactctttccctacacgacgctcttccgatctgcataacttccataccaaggttagcacgg
LspsbA R	gtgactggagttcagacgtgtgctcttccgatctcatggttattttggccgattgatcttcc
LpsaA F	acactctttccctacacgacgctcttccgatctcccggtctagcccattcctcgaatgaag
LpsaA R	gtgactggagttcagacgtgtgctcttccgatctctctagtgaataactaaagaaaatagatagatgggag

Supplementary Sequence 1. Amino acid sequences of TALED components.

CTS
MDSQLVLSLKLNPSFTPLSPLFPFTPCSSFSPSLRFSSCYSRRLYSPVTVYAAK

3xflag
DYKDHDGDYKDHDIDYKDDDDK

N terminal domain of TALE array
DLRTLGYSQQQQEKIKPKVRSTVAQHHEALVGHGFTHAHIVALSQHPAALGTVAVKYQDMIAALPEATHE
AIVGVGKQWSGARALEALLTVAGELRGPPLQLDTGQLLKIAKRGGVTAVEAVHAWRNALTGAPLN

C terminal domain of TALE array
LTPEQVVAIASNGGGKQALESIVAQLSRPDPALAALTNDHLVALACLGGRPALDAVKKGLG

Linker 1 (TALE array – Linker 1-DddA$_{tox}$)
GS

Linker 2 (DddA$_{tox}$-Linker 2 -ABE8.0)
SGSETPGTSESATPES

DddA$_{tox}$ 1397N
GSGSYALGPYQISAPQLPAYNGQTVGTFYYVNDAGGLESKVFSSGGPTPYPNYANAGHVEGQSALFMRD
NGISEGLVFHNNPEGTCGFCVNMTETLLPENAKMTVVPPEG

DddA$_{tox}$ 1397C
GSAIPVKRGATGETKVFTGNSNSPKSPTKGGC

ABE 8.0
SEVEFSHEYWMRHALTLAKRARDEREVPVGAVLVLNNRVIGEGWNRAIGLHDPTAHAEIMALRQGGLV
MQNYRLIDATLYVTFEPCVMCAGAMIHSRIGRVVFGVRNSKRGAAGSLMNVLNYPGMNHRVEITEGILA
DECAALLCDFYRMPRQVFNAQKKAQSSIN

psbA Left TALE repeat
LTPAQVVAIASNGGGKQALETVQRLLPVLCQAHGLTPAQVVAIASNNGGKQALETVQRLLPVLCQDHGLT
PAQVVAIASNIGGKQALETVQRLLPVLCQDHGLTPDQVVAIASNIGGKQALETVQRLLPVLCQAHGLTPDQ
VVAIASNIGGKQALETVQRLLPVLCQDHGLTPDQVVAIASNGGGKQALETVQRLLPVLCQAHGLTPAQVV
AIASNGGGKQALETVQRLLPVLCQAHGLTPAQVVAIASNNGGKQALETVQRLLPVLCQDHGLTPAQVVAI
ASNIGGKQALETVQRLLPVLCQAHGLTPDQVVAIASNIGGKQALETVQRLLPVLCQAHGLTPDQVVAIASN
IGGKQALETVQRLLPVLCQAHGLTPAQVVAIASHDGGKQALETVQRLLPVLCQAHGLTPAQVVAIASHDG

GKQALETVQRLLPVLCQDHGLTPDQVVAIASNIGGKQALETVQRLLPVLCQDHGLTPAQVVAIASNGGGK
QALETVQRLLPVLCQAHGLTPEQVVAIASNGGGKQALETVQRLLPVLCQAHGLTPDQVVAIASNGGGKQA
LETVQRLLPVLCQAHG

psbA Right TALE repeat

LTPDQVVAIASNNGGKQALETVQRLLPVLCQAHGLTPAQVVAIASHDGGKQALETVQRLLPVLCQDHGLT
PDQVVAIASNGGGKQALETVQRLLPVLCQAHGLTPEQVVAIASNGGGKQALETVQRLLPVLCQAHGLTPD
QVVAIASNGGGKQALETVQRLLPVLCQDHGLTPDQVVAIASNIGGKQALETVQRLLPVLCQDHGLTPAQV
VAIASNNGGKQALETVQRLLPVLCQDHGLTPAQVVAIASNNGGKQALETVQRLLPVLCQDHGLTPDQVV
AIASNGGGKQALETVQRLLPVLCQAHGLTPDQVVAIASNIGGKQALETVQRLLPVLCQDHGLTPAQVVAIA
SNGGGKQALETVQRLLPVLCQAHGLTPAQVVAIASHDGGKQALETVQRLLPVLCQDHGLTPAQVVAIASN
IGGKQALETVQRLLPVLCQDHGLTPDQVVAIASNNGGKQALETVQRLLPVLCQDHGLTPEQVVAIASHDG
GKQALETVQRLLPVLCQAHGLTPEQVVAIASNIGGKQALETVQRLLPVLCQAHGLTPAQVVAIASHDGGK
QALETVQRLLPVLCQDHG

rrn16S Left TALE repeat

LTPAQVVAIASNIGGKQALETVQRLLPVLCQDHGLTPDQVVAIASNIGGKQALETVQRLLPVLCQDHGLTP
DQVVAIASNNGGKQALETVQRLLPVLCQAHGLTPAQVVAIASNNGGKQALETVQRLLPVLCQAHGLTPD
QVVAIASNNGGKQALETVQRLLPVLCQDHGLTPEQVVAIASNNGGKQALETVQRLLPVLCQAHGLTPAQ
VVAIASHDGGKQALETVQRLLPVLCQDHGLTPDQVVAIASNIGGKQALETVQRLLPVLCQDHGLTPAQVV
AIASNGGGKQALETVQRLLPVLCQAHGLTPAQVVAIASNNGGKQALETVQRLLPVLCQDHGLTPAQVVAI
ASNIGGKQALETVQRLLPVLCQAHGLTPEQVVAIASNGGGKQALETVQRLLPVLCQAHGLTPAQVVAIAS
NNGGKQALETVQRLLPVLCQDHGLTPAQVVAIASNIGGKQALETVQRLLPVLCQAHGLTPDQVVAIASHD
GGKQALETVQRLLPVLCQAHGLTPEQVVAIASNGGGKQALETVQRLLPVLCQAHGLTPAQVVAIASNGGG
KQALETVQRLLPVLCQAHGLTPDQVVAIASNNGGKQALETVQRLLPVLCQDHG

rrn16S Right TALE repeat

LTPDQVVAIASNNGGKQALETVQRLLPVLCQAHGLTPDQVVAIASHDGGKQALETVQRLLPVLCQDHGLT
PDQVVAIASHDGGKQALETVQRLLPVLCQAHGLTPAQVVAIASNNGGKQALETVQRLLPVLCQDHGLTPA
QVVAIASNNGGKQALETVQRLLPVLCQDHGLTPDQVVAIASNGGGKQALETVQRLLPVLCQAHGLTPAQ
VVAIASNNGGKQALETVQRLLPVLCQDHGLTPAQVVAIASNIGGKQALETVQRLLPVLCQAHGLTPEQVV
AIASNGGGKQALETVQRLLPVLCQAHGLTPAQVVAIASNIGGKQALETVQRLLPVLCQDHGLTPDQVVAIA
SNIGGKQALETVQRLLPVLCQDHGLTPDQVVAIASNNGGKQALETVQRLLPVLCQAHGLTPAQVVAIASH
DGGKQALETVQRLLPVLCQDHGLTPDQVVAIASHDGGKQALETVQRLLPVLCQAHGLTPEQVVAIASNN
GGKQALETVQRLLPVLCQAHGLTPDQVVAIASNNGGKQALETVQRLLPVLCQDHGLTPAQVVAIASNIGG
KQALETVQRLLPVLCQAHGLTPEQVVAIASNNGGKQALETVQRLLPVLCQAHG

psaA Left TALE repeat

LTPAQVVAIASHDGGKQALETVQRLLPVLCQAHGLTPAQVVAIASNGGGKQALETVQRLLPVLCQDHGLT
PDQVVAIASNNGGKQALETVQRLLPVLCQDHGLTPAQVVAIASNNGGKQALETVQRLLPVLCQDHGLTPE
QVVAIASNGGGKQALETVQRLLPVLCQAHGLTPAQVVAIASNGGGKQALETVQRLLPVLCQDHGLTPAQV
VAIASHDGGKQALETVQRLLPVLCQDHGLTPDQVVAIASHDGGKQALETVQRLLPVLCQAHGLTPEQVVA
IASNNGGKQALETVQRLLPVLCQAHGLTPAQVVAIASNNGGKQALETVQRLLPVLCQAHGLTPDQVVAIA
SHDGGKQALETVQRLLPVLCQDHGLTPAQVVAIASNNGGKQALETVQRLLPVLCQAHGLTPDQVVAIAS
NIGGKQALETVQRLLPVLCQAHGLTPDQVVAIASNIGGKQALETVQRLLPVLCQAHGLTPAQVVAIASHDG
GKQALETVQRLLPVLCQAHGLTPEQVVAIASNNGGKQALETVQRLLPVLCQAHGLTPDQVVAIASNIGGK
QALETVQRLLPVLCQDHGLTPDQVVAIASNIGGKQALETVQRLLPVLCQAHG

psaA Right TALE repeat

LTPEQVVAIASNNGGKQALETVQRLLPVLCQAHGLTPDQVVAIASNGGGKQALETVQRLLPVLCQDHGLT
PAQVVAIASNNGGKQALETVQRLLPVLCQAHGLTPEQVVAIASNGGGKQALETVQRLLPVLCQAHGLTPA
QVVAIASNGGGKQALETVQRLLPVLCQAHGLTPDQVVAIASNNGGKQALETVQRLLPVLCQDHGLTPAQ
VVAIASNGGGKQALETVQRLLPVLCQDHGLTPAQVVAIASHDGGKQALETVQRLLPVLCQAHGLTPEQVV
AIASHDGGKQALETVQRLLPVLCQAHGLTPAQVVAIASNNGGKQALETVQRLLPVLCQAHGLTPDQVVAI
ASNNGGKQALETVQRLLPVLCQDHGLTPAQVVAIASNIGGKQALETVQRLLPVLCQAHGLTPAQVVAIAS
NIGGKQALETVQRLLPVLCQDHGLTPDQVVAIASNIGGKQALETVQRLLPVLCQDHGLTPDQVVAIASNN
GGKQALETVQRLLPVLCQAHGLTPAQVVAIASNIGGKQALETVQRLLPVLCQAHGLTPAQVVAIASNNGG
KQALETVQRLLPVLCQDHGLTPAQVVAIASNNGGKQALETVQRLLPVLCQAHG

rbc L Left TALE repeat

LTPDQVVAIASNNGGKQALETVQRLLPVLCQAHGLTPDQVVAIASHDGGKQALETVQRLLPVLCQDHGLT
PDQVVAIASHDGGKQALETVQRLLPVLCQAHGLTPDQVVAIASNNGGKQALETVQRLLPVLCQDHGLTP
AQVVAIASNIGGKQALETVQRLLPVLCQAHGLTPEQVVAIASNNGGKQALETVQRLLPVLCQAHGLTPAQ
VVAIASNIGGKQALETVQRLLPVLCQDHGLTPAQVVAIASNGGGKQALETVQRLLPVLCQDHGLTPDQVV
AIASNIGGKQALETVQRLLPVLCQAHGLTPEQVVAIASNIGGKQALETVQRLLPVLCQAHGLTPAQVVAIAS
NGGGKQALETVQRLLPVLCQAHGLTPAQVVAIASNNGGKQALETVQRLLPVLCQDHGLTPDQVVAIASN
NGGKQALETVQRLLPVLCQAHGLTPDQVVAIASHDGGKQALETVQRLLPVLCQDHGLTPDQVVAIASHD
GGKQALETVQRLLPVLCQAHGLTPEQVVAIASNGGGKQALETVQRLLPVLCQAHGLTPDQVVAIASNIGG
KQALETVQRLLPVLCQAHGLTPAQVVAIASHDGGKQALETVQRLLPVLCQDHG

rbc L Right TALE repeat

LTPDQVVAIASNIGGKQALETVQRLLPVLCQDHGLTPAQVVAIASNGGGKQALETVQRLLPVLCQAHGLTP
AQVVAIASHDGGKQALETVQRLLPVLCQDHGLTPDQVVAIASNIGGKQALETVQRLLPVLCQAHGLTPDQ
VVAIASNIGGKQALETVQRLLPVLCQDHGLTPDQVVAIASNGGGKQALETVQRLLPVLCQAHGLTPDQVV
AIASNIGGKQALETVQRLLPVLCQAHGLTPDQVVAIASNIGGKQALETVQRLLPVLCQAHGLTPAQVVAIA
SHDGGKQALETVQRLLPVLCQAHGLTPAQVVAIASNIGGKQALETVQRLLPVLCQDHGLTPDQVVAIASN
NGGKQALETVQRLLPVLCQDHGLTPEQVVAIASHDGGKQALETVQRLLPVLCQAHGLTPEQVVAIASNNG

GKQALETVQRLLPVLCQAHGLTPDQVVAIASNGGGKQALETVQRLLPVLCQDHGLTPAQVVAIASNNGG KQALETVQRLLPVLCQAHGLTPAQVVAIASHDGGKQALETVQRLLPVLCQDHGLTPDQVVAIASNIGGKQ ALETVQRLLPVLCQDHGLTPAQVVAIASNGGGKQALETVQRLLPVLCQAHG

Supplementary Sequence 2. DNA sequences of RPS5A promoter and 35S terminator.

RPS5A promoter

CTCAACTTTTGATTCGCTATTTGCAGTGCACCTGTGGCGTTCATCACATCTTTTGTGACACTGTTTGCACT
GGTCATTGCTATTACAAAGGACCTTCCTGATGTTGAAGGAGATCGAAAGTAAGTAACTGCACGCATAAC
CATTTTCTTTCCGCTCTTTGGCTCAATCCATTTGACAGTCAAAGACAATGTTTAACCAGCTCCGTTTGATA
TATTGTCTTTATGTGTTTGTTCAAGCATGTTTAGTTAATCATGCCTTTGATTGATCTTGAATAGGTTCCAAA
TATCAACCCTGGCAACAAAACTTGGAGTGAGAAACATTGCATTCCTCGGTTCTGGACTTCTGCTAGTAAA
TTATGTTTCAGCCATATCACTAGCTTTCTACATGCCTCAGGTGAATTCATCTATTTCCGTCTTAACTATTTCG
GTTAATTAAAGCACGAACACCATTACTGCATGTAGAAGCTTGATAAACTATCGCCACCAATTTATTTTTGT
TGCGATATTGTTACTTTCCTCAGTATGCAGCTTTGAAAAGACCAACCCTCTTATCCTTTAACAATGAACAG
GTTTTTAGAGGTAGCTTGATGATTCCTGCACATGTGATCTTGGCTTCAGGCTTAATTTTCCAGGTAAAGC
ATTATGAGATACTCTTATATCTCTTACATACTTTTGAGATAATGCACAAGAACTTCATAACTATATGCTTTA
GTTTCTGCATTTGACACTGCCAAATTCATTAATCTCTAATATCTTTGTTGTTGATCTTTGGTAGACATGGGT
ACTAGAAAAAGCAAACTACACCAAGGTAAAATACTTTTGTACAAACATAAACTCGTTATCACGGAACAT
CAATGGAGTGTATATCTAACGGAGTGTAGAAACATTTGATTATTGCAGGAAGCTATCTCAGGATATTATC
GGTTTATATGGAATCTCTTCTACGCAGAGTATCTGTTATTCCCCTTCCTCTAGCTTTCAATTTCATGGTGAG
GATATGCAGTTTTCTTTGTATATCATTCTTCTTCTTCTTTGTAGCTTGGAGTCAAAATCGGTTCCTTCATGT
ACATACATCAAGGATATGTCCTTCTGAATTTTTATATCTTGCAATAAAAATGCTTGTACCAATTGAAACAC
CAGCTTTTTGAGTTCTATGATCACTGACTTGGTTCTAACCAAAAAAAAAAAAAATGTTTAATTTACATATCT
AAAAGTAGGTTTAGGGAAACCTAAACAGTAAAATATTTGTATATTATTCGAATTTCACTCATCATAAAAA
CTTAAATTGCACCATAAAATTTTGTTTTACTATTAATGATGTAATTTGTGTAACTTAAGATAAAAATAATAT
TCCGTAAGTTAACCGGCTAAAACCACGTATAAACCAGGGAACCTGTTAAACCGGTTCTTTACTGGATAAA
GAAATGAAAGCCCATGTAGACAGCTCCATTAGAGCCCAAACCCTAAATTTCTCATCTATATAAAAGGAGT
GACATTAGGGTTTTTGTTCGTCCTCTTAAAGCTTCTCGTTTTCTCTGCCGTCTCTCTCATTCGCGCGACGCA
AACGATCTTCAGGTGATCTTCTTTCTCCAAATCCTCTCTCATAACTCTGATTTCGTACTTGTGTATTTGAGC
TCACGCTCTGTTTCTCTCACCACAGCC

35S terminator

CGGCCATGCTAGAGTCCGCAAAAATCACCAGTCTCTCTCTACAAATCTATCTCTCTCTATTTTTCTCCAGA
ATAATGTGTGAGTAGTTCCCAGATAAGGGAATTAGGGTTCTTATAGGGTTTCGCTCATGTGTTGAGCATAT
AAGAAACCCTTAGTATGTATTTGTATTTGTAAAATACTTCTATCAATAAAATTTCTAATTCCTAAAACCAA
AATCCAGTGACCT
