# Supplementary material for: Targeted A-to-G base editing of chloroplast DNA in plants
Source: Nat Plants. 2022 Dec 1;8(12):1378–84. doi: 10.1038/s41477-022-01279-8 (PMC9788985; doi:10.1038/s41477-022-01279-8)
Supplement: Supplementary file 2 — Reporting Summary [file 41477_2022_1279_MOESM2_ESM.pdf]

nature portfolio

Corresponding author(s): Jin-Soo Kim

Last updated by author(s): Oct. 12, 2022

Reporting Summary

Nature Portfolio wishes to improve the reproducibility of the work that we publish. This form provides structure for consistency and transparency in reporting. For further information on Nature Portfolio policies, see our Editorial Policies and the Editorial Policy Checklist.

Statistics

For all statistical analyses, confirm that the following items are present in the figure legend, table legend, main text, or Methods section.

n/a	Confirmed	
☐	☒	The exact sample size (*n*) for each experimental group/condition, given as a discrete number and unit of measurement
☐	☒	A statement on whether measurements were taken from distinct samples or whether the same sample was measured repeatedly
☒	☐	The statistical test(s) used AND whether they are one- or two-sided<br>*Only common tests should be described solely by name; describe more complex techniques in the Methods section.*
☒	☐	A description of all covariates tested
☒	☐	A description of any assumptions or corrections, such as tests of normality and adjustment for multiple comparisons
☐	☒	A full description of the statistical parameters including central tendency (e.g. means) or other basic estimates (e.g. regression coefficient) AND variation (e.g. standard deviation) or associated estimates of uncertainty (e.g. confidence intervals)
☒	☐	For null hypothesis testing, the test statistic (e.g. *F*, *t*, *r*) with confidence intervals, effect sizes, degrees of freedom and *P* value noted<br>*Give P values as exact values whenever suitable.*
☒	☐	For Bayesian analysis, information on the choice of priors and Markov chain Monte Carlo settings
☒	☐	For hierarchical and complex designs, identification of the appropriate level for tests and full reporting of outcomes
☒	☐	Estimates of effect sizes (e.g. Cohen's *d*, Pearson's *r*), indicating how they were calculated

Our web collection on statistics for biologists contains articles on many of the points above.

Software and code

Policy information about availability of computer code

Data collection	The MiniSeq system (Illumina) was used to perform targeted deep sequencing. The HiSeq X Ten platform (Illumina) was used whole chloroplast genome sequencing.
Data analysis	Source code (https://github.com/ibs-cge/maund) was used to determine based editing frequencies from targeted deep sequencing data. For data analysis and visualization, we used BWA (v.0.7.17), pysam (v0.18.0), GraphPad Prism 8, Microsoft Excel 2016, PowerPoint 2016, and Adobe Illustrator CS6.

For manuscripts utilizing custom algorithms or software that are central to the research but not yet described in published literature, software must be made available to editors and reviewers. We strongly encourage code deposition in a community repository (e.g. GitHub). See the Nature Portfolio guidelines for submitting code & software for further information.

Data

Policy information about availability of data

All manuscripts must include a data availability statement. This statement should provide the following information, where applicable:

- Accession codes, unique identifiers, or web links for publicly available datasets
- A description of any restrictions on data availability
- For clinical datasets or third party data, please ensure that the statement adheres to our policy

All data supporting the findings of this study are available in the paper, Extended Data and Supplementary Information. Source data are provided with this paper. DNA sequencing data were deposited in the Sequence Read Archive (SRA) database of the National Center for Biotechnology Information (NCBI) with the BioProject accession code PRJNA858174. BioProject accession code is release on Oct. 12, 2022.

Field-specific reporting

Please select the one below that is the best fit for your research. If you are not sure, read the appropriate sections before making your selection.

☒ Life sciences ☐ Behavioural & social sciences ☐ Ecological, evolutionary & environmental sciences

For a reference copy of the document with all sections, see nature.com/documents/nr-reporting-summary-flat.pdf

Life sciences study design

All studies must disclose on these points even when the disclosure is negative.

Sample size	For transfection experiments, approximately 500,000 lettuce protoplasts were used in each experiment. At least 63 T1 plants and 37 T2 plants for each targeted genes were analyzed by targeted deep sequencing. No statistical method was used to predetermine sample size. These sample size was maximum number of plants that we could handle in the growth rooms.
Data exclusions	No data were excluded from the analyses.
Replication	The protoplasts experiments were performed from individual biological replicates (n=3). All attempts at replication were successful.
Randomization	Protoplast samples were randomly collected from a large pool of lettuce protoplasts for each transfection.
Blinding	Blinding was not required because all analyses including targeted deep sequencing and phenotyping by antibiotics and molecular methods could be carried out without making any subjective judgements.

Reporting for specific materials, systems and methods

We require information from authors about some types of materials, experimental systems and methods used in many studies. Here, indicate whether each material, system or method listed is relevant to your study. If you are not sure if a list item applies to your research, read the appropriate section before selecting a response.

Materials & experimental systems

n/a	Involved in the study	
☒	☐	Antibodies
☒	☐	Eukaryotic cell lines
☒	☐	Palaeontology and archaeology
☒	☐	Animals and other organisms
☒	☐	Human research participants
☒	☐	Clinical data
☒	☐	Dual use research of concern

Methods

n/a	Involved in the study	
☒	☐	ChIP-seq
☒	☐	Flow cytometry
☒	☐	MRI-based neuroimaging
